# Ion Channel–Extracellular Matrix Interplay in Colorectal Cancer: A Network-Based Approach to Tumor Microenvironment Remodeling

**DOI:** 10.3390/ijms26115147

**Published:** 2025-05-27

**Authors:** Alberta Terzi, Fatima Maqoud, Davide Guido, Domenica Mallardi, Michelangelo Aloisio, Blendi Ura, Nicolò Gualandi, Francesco Russo, Gianluigi Giannelli

**Affiliations:** 1 Unit of Personalized Medicine, National Institute of Gastroenterology IRCCS “Saverio de Bellis”, Castellana Grotte, 70013 Bari, Italy; alberta.terzi@irccsdebellis.it; 2 Functional Gastrointestinal Disorders Research Group, National Institute of Gastroenterology IRCCS “Saverio de Bellis”, Castellana Grotte, 70013 Bari, Italy; domenica.mallardi@irccsdebellis.it (D.M.); michelangelo.aloisio@irccsdebellis.it (M.A.); 3 Unit of Data Science, National Institute of Gastroenterology IRCCS “Saverio de Bellis”, Castellana Grotte, 70013 Bari, Italy; davide.guido@irccsdebellis.it; 4 Institute for Maternal and Child Health-IRCCS “Burlo Garofolo”, 34137 Trieste, Italy; blendi.ura@burlo.trieste.it; 5 Laboratory of Biochemistry, Department of Medicine, University of Udine, P.le Kolbe 4, 33100 Udine, Italy; nicolo.gualandi@uniud.it; 6 Scientific Direction, National Institute of Gastroenterology IRCCS “Saverio de Bellis”, Castellana Grotte, 70013 Bari, Italy; gianluigi.giannelli@irccsdebellis.it

**Keywords:** colorectal cancer, ion channels, extracellular matrix, transcriptomics, causal network inference

## Abstract

The progression of colorectal cancer (CRC) is driven by dynamic interactions between tumor cells and their microenvironment, particularly the extracellular matrix (ECM). Ion channels, critical regulators of cellular signaling, have emerged as mediators of ECM remodeling and tumor aggressiveness. In this study, we integrate transcriptomic data from 185 CRC tumors and 157 adjacent normal tissues with network modeling to dissect the interplay between ion channels and the ECM. We identified 4036 differentially expressed genes (DEGs), including 188 ion channel-associated DEGs (IC-DEGs) enriched in ECM-related pathways, such as collagen assembly, matrix metalloproteinase regulation, and mechanotransduction. Structural equation modeling revealed an active CRC−ion channel module (CRC-IC) comprising 482 nodes and 422 edges, highlighting dysregulated interactions between ECM components (e.g., *COL1A1*, *COL5A2*, *VCAN*, *LAMA4*, *LA-MA5*, *LAMC1*), ion channels (e.g., *TRPM5* and *SLC16A1*), and cytoskeletal regulators. Key nodes, including *CHST11* and *VCAN*, were associated with ECM sulfation, tumor invasiveness, and immune evasion. Notably, survival was associated with *MAPK1*, *SLC16A1*, and *ABCB4* in relation to patient prognosis. Our findings underscore the pivotal role of ion channels as co-factors in ECM dynamics in CRC, offering mechanistic insights into tumor-stroma crosstalk and identifying potential therapeutic targets to disrupt microenvironment-driven progression.

## 1. Introduction

CRC is one of the most prevalent and lethal malignancies worldwide [1] and is characterized by extensive molecular and cellular heterogeneity that drives tumor initiation, progression, and metastasis. While genetic mutations in key oncogenic pathways, including WNT/β-catenin, TP53, and KRAS, are well-established drivers of CRC [2,3], mounting evidence highlights the pivotal role of the tumor microenvironment (TME) in shaping disease pathophysiology [4]. Among its critical components, the ECM plays a central role in modulating tumor-cell behavior. The TME has gained prominence recently, highlighting how its composition and architecture provide biochemical and biomechanical cues that regulate proliferation, invasion, and therapy resistance. Tumors are characterized by the uncontrolled growth of epithelial cells and alterations in the surrounding stromal compartment. Physiologically, the relationship between fibroblasts and ECM is termed “mechanoreciprocity”, which relies on the ability of cells to sense physical cues from their environment, such as stiffness and mechanical stress, and to subsequently adjust their morphology and behavior in response to these stimuli. Cells can modify the ECM by secreting new components, influencing the matrix structure and stiffness. The composition and organization of the ECM influence cytoskeletal tension, prompting cells to contract and remodel their surrounding matrix until they reach “tension homeostasis”, that is, mechanical equilibrium. This mechanism is dysregulated during pathological stiffening of the stroma, leading to fibrosis or desmoplasia, that is, fibrotic tissue over-deposition, typical of malignant solid tumors. In CRC, this process is characterized by altered deposition and remodeling of the ECM, where collagen fibers, fibronectin, vitronectin, osteopontin, tenascin C, and polysaccharides are compactly and densely deposited [5]. These ECM alterations are closely linked to patient prognosis and therapeutic outcomes.

Beyond the ECM, ion channels are emerging as key regulators of CRC progression, acting as mediators of ECM remodeling and interfacing with critical signaling networks that sustain tumor growth and metastatic competence.

Ion channels are integral membrane proteins that regulate the movement of ions across the cell membrane. They are essential for many physiological processes, such as controlling cell volume, supporting migration, and enabling signal transduction [6]. In CRC, abnormal expression and function of several ion channels, like voltage-gated, ligand-gated, and mechanosensitive channels, have been linked to tumor progression [7,8,9]. These channels function as mechanotransducers, detecting alterations in ECM stiffness and composition and simultaneously modulating downstream signaling pathways that control cytoskeletal dynamics and cell adhesion [10]. Consequently, ion channel-mediated cation flux influences diverse cellular responses, including growth, migration, adhesion, morphogenesis, gene expression, fluid homeostasis and vesicular transport. Dysregulated ion channel activity has been linked to epithelial-mesenchymal transition (EMT), cellular homeostasis disruption, and invasive phenotypes promotion, underscoring its importance in CRC pathophysiology [11,12].

Ion channels and ECM components engage in reciprocal regulatory interactions. ECM remodeling enzymes, such as matrix metalloproteinases (MMPs), and mechanical cues induced by matrix stiffening modulate ion channel activity by altering the biochemical microenvironment and releasing bioactive factors that influence channel function [13,14,15]. Conversely, ion channels regulate ECM integrity by controlling intracellular calcium signaling, pH homeostasis, and the secretion of proteolytic enzymes, thereby influencing collagen deposition, fibronectin assembly, and the overall ECM architecture [16,17]. This bidirectional communication establishes a dynamic feedback loop that sustains CRC progression and contributes to chemoresistance by promoting a protective stromal niche [18]. The desmoplastic context of CRC creates a tumor environment characterized by hypoxia, acidic pH, and mechanical alterations to the ECM network, which further affect the expression and function of ion channels and ECM remodeling. These aberrant changes promote therapy resistance and enhance cancer aggression [19,20].

Recent advances in systems biology have highlighted the potential of network-based approaches for deciphering complex biological systems [21]. Graph theory and structural equation modeling (SEM) [22,23] are powerful tools for exploring transcriptomic interactions, identifying active disease modules, and inferring functional effects within complex molecular networks. In the context of CRC, these approaches can clarify the interplay between ion channels and the ECM, offering insights into the mechanisms that underlie tumor progression and therapy resistance. By integrating transcriptomic data with network models, it is possible to uncover modular structures and community interactions that reflect the functional architecture of CRC.

Given the (i) complex and multifactorial nature of CRC, driven by both genetic and environmental factors, (ii) emerging role of ion channels as key mediators of tumor progression and ECM remodeling, and (iii) reciprocal regulatory interactions between ion channels and the ECM, a causal network-based approach utilizing graph theory [24,25] and structural equation modeling [26] could be highly beneficial. This approach enables the exploration of transcriptomic interactions, such as active disease modules and their associated communities, and facilitates inferences regarding their functional effects.

This study aimed to explore the transcriptomic complexity of ion channel networks in CRC, focusing on their interactions with the ECM. We analyzed these complex interactions using graph theory and SEM and inferred their functional effects. Our approach integrates gene expression data with molecular interaction databases, such as Reactome, to enhance understanding of disease mechanisms. By identifying pathways underlying disease-specific expression patterns, we discovered “hidden node” genes—non-differentially expressed genes that are functionally critical for network connectivity. This revealed a CRC-specific disease module and a network of disrupted cellular components that potentially drive tumor progression. Using network modeling, we further characterized a CRC-IC and its functional communities, clarifying their roles in CRC. This innovative strategy provides novel insights into the molecular mechanisms governing ion channel−ECM interactions in CRC, their impact on the desmoplastic reaction within the “in situ” tumor, and their roles in metastasis and pathological progression.

## 2. Results

### 2.1. Dataset Description and Quality Control (QC)

The study analyzed a comprehensive RNA sequencing (RNA-seq) dataset generated from primary CRC samples and matched Normal Adjacent Tissues (NAT) obtained from a Korean cohort (*n* = 185) [27]. All samples were obtained from patients who did not receive neoadjuvant chemotherapy, as this treatment can significantly alter the gene expression profiles [27]. This ensures that the observed transcriptomic differences are primarily driven by the intrinsic characteristics of CRC rather than treatment-induced changes.

The dataset comprises 56,609 features (genes) across 342 samples, including 185 primary CRC tumor samples and 157 NAT samples. All samples passed the coverage quality control as they exceeded the threshold of 25 million reads, with a range of 25.8 to 186.8 million reads per sample (Table S1). The database comprises 185 patients with a mean age of 62.3 ± 10.5 years, of whom 60.5% were males and 39.5% were females; most presented with grade 3 CRC. All samples were confirmed to be adenocarcinomas (Table 1). The extensive sequencing depth ensures the robust detection of highly expressed and low-abundance transcripts, providing a solid foundation for differential expression analysis and downstream investigations.

### 2.2. CRC DEGs Determination and Ion Channel Subset

Preliminary analysis using Principal Component Analysis (PCA) revealed that disease status accounted for 48% of the transcriptomic variability (PC1), indicating a clear separation between tumor and normal tissues (Figure S1A). Sex accounted for 6% of the variability (PC2), suggesting a secondary but notable influence on gene expression patterns (Figure S1B). Notably, disease grade did not contribute significantly to the remaining variability (Figure S1C), suggesting that other factors may be more prominent in shaping this landscape.

**Figure 1:**
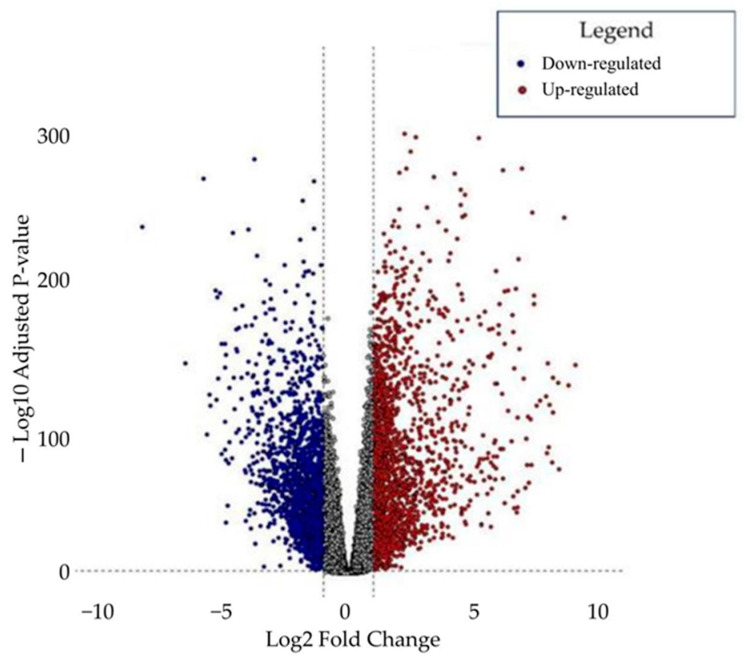
Volcano plot. A total of 4036 DEGs were identified, including 2050 downregulated (blue) and 1986 upregulated (red) genes, with an adjusted *p*-value < 0.01. Vertical dashed lines mark the log_2_ Fold Change thresholds of ±1 (up- and downregulated, respectively), and the horizontal dashed line indicates the significance threshold of −log_10_(adjusted *p*-value) = 2 (*p* < 0.01).

Differential expression analysis was performed to investigate transcriptomic complexity in CRC further by comparing 185 CRC samples with 157 NAT samples. DEGs were identified using stringent criteria, including an adjusted *p*-value < 0.01 and a log2 Fold Change threshold of ≥1 for upregulated genes and ≤−1 for downregulated genes. This analysis identified 4036 DEGs, of which 1986 were upregulated and 2050 were downregulated. The complete list of DEGs is provided in Table S2, with their expression patterns visualized as a volcano plot (Figure 1).

The ion-channel panel, comprising 720 genes, was curated from the NCBI Gene database [https://www.ncbi.nlm.nih.gov/gene/ (accessed on 3 March 2025)] using specific search terms and filters to ensure comprehensive coverage of genes related to ion channels. The entire panel is presented in Table S3.

**Figure 2:**
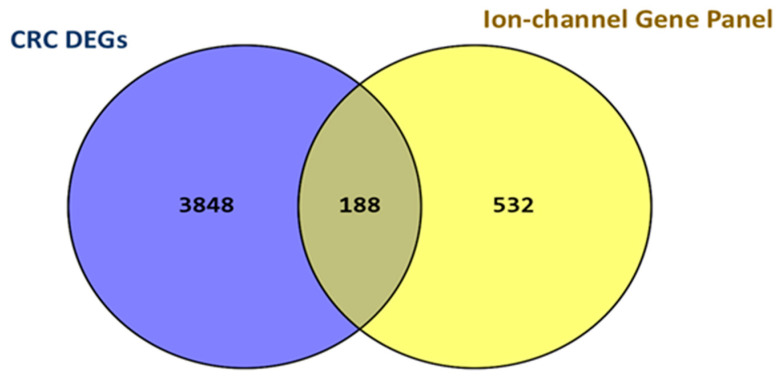
Identification of IC-DEGs in CRC. A total of 188 IC-DEGs were identified by intersecting the 4036 CRC DEGs with a panel of 720 ion-channel genes using Venny v2.1, https://bioinfogp.cnb.csic.es/tools/venny/index.html (accessed on 5 March 2025).

A systematic intersection of CRC DEGs with the ion-channel panel genes revealed 188 IC-DEGs that exhibited significantly differential expression (Figure 2, Table S4). This subset suggests a potential contribution of ion channel dysregulation to the molecular characteristics of CRC. The identified genes span diverse ion channel families, including voltage-gated, ligand-gated, and mechanosensitive channels, underscoring the involvement of multiple ion transport and signaling mechanisms in CRC pathophysiology.

### 2.3. Reactome Enrichment Analysis Results

**Figure 3:**
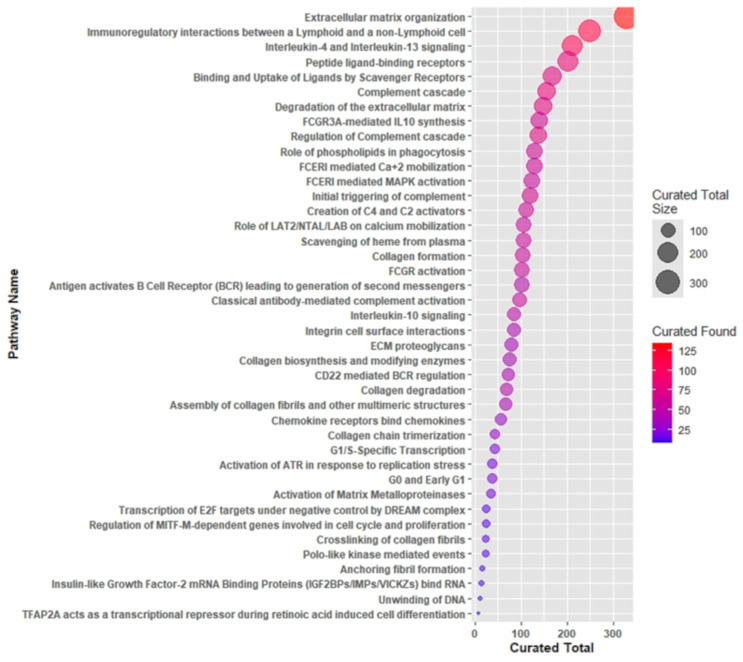
Reactome pathways identified by the 188 IC-DEGs. The dots represent the pathways, with dot size indicating the total number of genes and color denoting the number of DEGs in the pathway.

The 188 IC-DEGs were subsequently analyzed using Reactome (v91) pathway enrichment tools. This analysis identified 396 pathways (Table S5), with the top 39 pathways highlighted in Figure 3. These pathways prominently emphasize the transport mechanisms and precise regulation of ion flux across membranes. Notably, key pathways included those involved in tight junction interactions, essential for maintaining the integrity of epithelial and endothelial barriers, the cellular response to bacterial toxins, and the mechanisms regulating the assembly of collagen fibrils and other ECM components. Moreover, WNT5A-dependent internalization of FZD4, along with non-integrin-mediated ECM interactions operated by receptors such as syndecans and CD44, have been identified as key regulators of cell adhesion and signaling, driving vital processes, including cell migration and differentiation. Approximately 40% of these pathways are directly or indirectly associated with ECM regulation, underscoring the fundamental role of ECM dynamics in CRC cell physiology. Additionally, the intricate relationship between ion channel activity and ECM processes, including synthesis, degradation, and remodeling, further emphasizes the significance of these pathways in CRC progression and the modulation of the tumor microenvironment.

### 2.4. IC-DEGs Validation in “The Cancer Genome Atlas” Database

To assess the consistency of our differential gene expression results, we conducted a comparative analysis between our dataset and publicly available transcriptomic data from The Cancer Genome Atlas (TCGA). We intersected our DEGs with genes encoding ion channels and analyzed their expression patterns using the KM Plotter platform (A5 Genetics Ltd., Und, Hungary). This analysis incorporated a comprehensive dataset comprising 2137 CRC tumor samples from 17 independent cohorts. Our results revealed a high degree of concordance, with approximately 85% of the IC-DEGs aligning with TCGA data, while only 15% showed discordant expression patterns (Table S6). The substantial overlap between our IC-DEGs data and TCGA results underscores the robustness and reproducibility of our sequencing-based quantification. Furthermore, this validation underscores the reliability of our gene expression profile in a Korean cohort comprising 185 primary CRC samples and 157 matched NAT samples. Moreover, this confirms the validity of the statistical method used. Overall, these results strengthen the translational significance of our dataset in elucidating the dysregulation of ion channel genes in colorectal cancer.

### 2.5. CRC-IC Module and Statistical Analysis

#### 2.5.1. CRC-IC Module Analysis

In the preliminary step, SEM-based Gene Set Analysis (SEMgsa) was conducted on notable genes, excluding the newly discovered transcripts identified through RNA-seq analysis. In the Supplemental Materials, a list of 4798 unique SEMgsa-DEGs is presented (Table S7). Consequently, these DEGs served as seed genes for the Steiner tree (ST) identification procedure.

CRC-IC ST was generated by integrating Reactome pathways [25] (considering the output at the intersection with ion channel genes) and data. The relative graph (Tables S8–S10) includes 581 nodes and 602 edges.

The active CRC-IC module, represented as the optimal Directed Acyclic Graph (DAG) (the beta coefficient was equal to 0.097), was derived using a fitting strategy based on SEM and graph theory, as described in the methods section and implemented in the R/SEM graph packages [27]. In this approach, we developed a data-driven model search strategy that relies solely on data without validation against a reference network. A total of 482 significant nodes (*p*-value < 0.05) and 422 significant edges were identified in the overall active module.

To better interpret the results of the SEM analysis, Tables S8–S10 in the Supplementary present the three final graphs of the active modules: one for CRC-IC samples, one for NAT control samples, and a combined “node and edge perturbation” model illustrating the differences between groups. These tables report significant direct effects (*p*-value < 0.05), with estimates > 0 representing upregulation effects and estimates < 0 indicating downregulation effects. Suggestive results (0.05 < *p* < 0.10) were also obtained. Additionally, effects involving groups indicated activation (if the estimate > 0) or inhibition (if the estimate < 0). Notably, no changes in the causal relationships were observed.

The standardized root mean-squared residual (SRMR) indexes were also provided to evaluate the goodness of fit: specifically, the overall model returned a SRMR = 0.192, the CRC-IC model a SRMR = 0.207, and the SRMR computed on NAT controls was 0.280.

**Figure 4:**
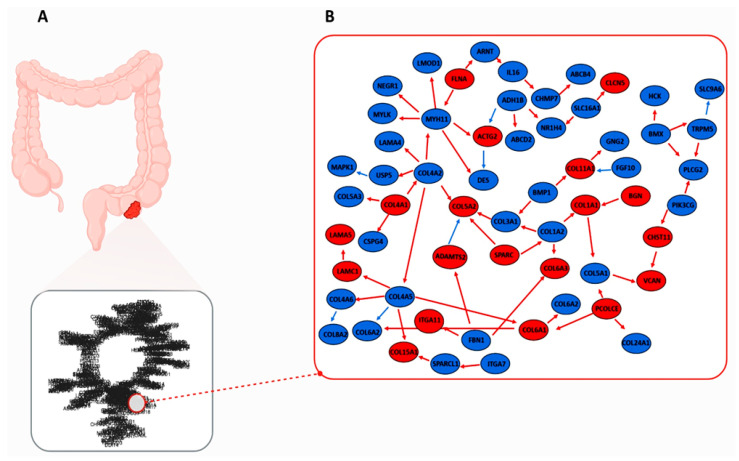
CRC-IC module and community. (**A**) Full CRC-IC module. The overall module obtained from the structural equation modeling (SEM) fitting is illustrated. (**B**) Reduced graph. Red nodes represent overexpressed genes, and blue nodes indicate downregulated genes. Red edges denote significant direct effects with a positive association (*p*-value < 0.05 and b effect > 0), and blue edges indicate significant direct effects with a negative association (*p*-value < 0.05 and b effect < 0).

In discussing our results, we chose not to display the ST plot and the final fitted plots (i.e., active modules) as figures, as the resolutions were too dense (available upon request from the authors). However, the results of the final fitted plots are presented in the tables above, due to the estimation of the one-to-one correspondence between the plots. To investigate the interaction between the ECM and ion channels in CRC in depth, we focused on a subset of the complete CRC-IC module, referred to as the reduced graph. This is illustrated in Figure 4 and consists of 58 significant nodes and 62 edges. The upregulated nodes numbered 19 with a *p*-value < 0.05, while the downregulated nodes totaled 39. The significantly upregulated edges were 54, and the downregulated edges were 8. The list of genes belonging to this sub-module can be found in Supplementary Table S11. Analysis of the reduced plot highlights a robust interaction between ion channels and ECM components in CRC. Notably, TRPM5 downregulation emerges as a key driver, exerting adverse effects on SLC9A6 while positively influencing PLCG2, ultimately leading to the overexpression of CHST11. This, in turn, contributes to tumor progression by modifying chondroitin sulfate and promoting VCAN upregulation. Additionally, ECM dysregulation is evident, characterized by the overexpression of key genes of the stromal and basal lamina components, such as COL5A2, COL1A1, COL4A1, COL6A3, LAMA5, LAMAC1, and COL15A1, alongside the upregulation of their activating enzymes, such as PCOLCE and ADAMT. Furthermore, downregulation of SLC16A1 is observed, which positively influences CLCN5, leading to its upregulation (Figure 4).

#### 2.5.2. Gene Communities Detection Results

Regarding the gene community structure identified through clustering, 36 topological clusters (modularity = 0.887) were identified in the optimal Directed Acyclic Graph (DAG). The Supplementary Materials (Table S12) provide a list indicating each gene’s cluster membership. Notably, the genes belonging to the reduced graph are part of the communities described in Table 2.

Table 2 summarizes the intersection between the identified gene communities and the reduced graph derived from the active CRC-IC module. Each row represents a specific community, providing the following details: Community_ID: Unique identifier for each gene community. Community gene number: The total number of genes belonging to the community. Intersection with reduced graph: The number of genes from the community present in the reduced graph. Intersection percentage: The proportion of genes from the community that overlap with the reduced graph, calculated as the ratio of “Intersection with reduced graph” to “Community gene number”, expressed as a percentage.

### 2.6. Characterization of CRC-IC Module Communities

**Figure 5:**
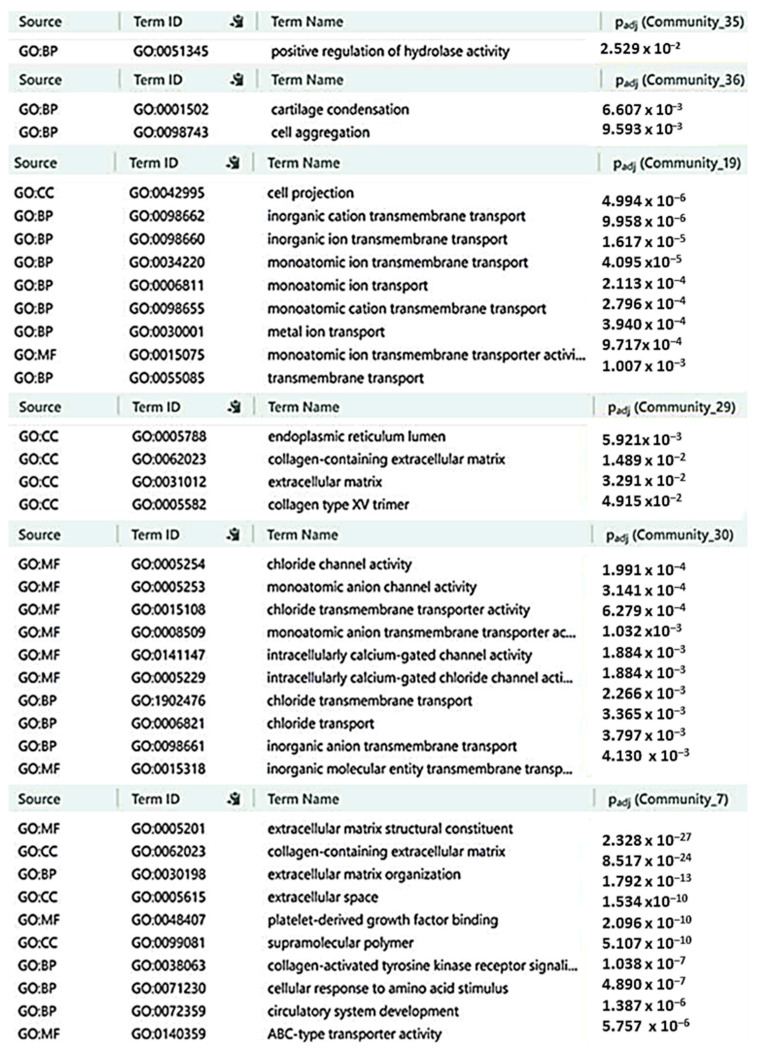
Functional enrichment analysis of communities linked to the CRC-IC reduced graph. The figure illustrates the top enriched Gene Ontology (GO) terms (Molecular Function: MF, Biological Process: BP, and Cellular Component: CC) and pathways for communities 35, 36, 19, 29, 30, and 7. These communities were selected based on their overlap exceeding 40% of their genes with the CRC-IC module’s reduced graph, emphasizing their critical role in the core module’s biological processes.

To further investigate the functional roles of the identified communities, we performed enrichment analyses using terms from the Gene Ontology (GO) database. The enrichment results for all 36 communities are provided in Supplementary Table S13. Figure 5 focuses on the six communities (7, 19, 29, 30, 35, and 36) with overlaps exceeding 40% of their genes with the reduced graph (Table 2). These communities were prioritized by ranking the adjusted *p*-values (from the enrichment analysis) and selecting the top 10 most statistically significant communities. However, only six of these top-ranked communities met the threshold of more than 40% overlap, indicating their strongest association with the core CRC-IC module and suggesting their central role in driving CRC-related biological processes. Enrichment analyses of communities 35 and 36 underscore the pivotal role of ion channels in modulating key biological pathways, including the positive regulation of hydrolase activity, cartilage condensation, and cell aggregation. These pathways are crucial for tumor progression, as highlighted by the influence of ion channels on cell-cell communication, ECM remodeling, and metastatic dissemination (Figure 5). In particular, calcium (Ca^2+^) and chloride (Cl^−^) channels play crucial roles in activating hydrolases, such as matrix metalloproteinases (MMPs), which promote ECM degradation and enhance tumor invasiveness. Voltage-gated Ca^2+^ channels (VGCCs) and store-operated calcium entry (SOCE) pathways amplify the intracellular signaling cascades that drive the activation of hydrolases, thereby enhancing CRC progression (Figure 5). Potassium (K^+^) channels, by modulating cell volume and mechanical properties, influence cell clustering and ECM-mediated signaling, mimicking cartilage condensation processes in the tumor microenvironment. Further pathway analyses from community 19 underscore the pivotal role of ion channels in regulating ion transport and intracellular signaling, both of which are crucial for maintaining cellular homeostasis and promoting oncogenic progression. Notably, investigations from community 29 identified critical ECM-associated pathways, including “collagen-containing extracellular matrix”, “extracellular matrix organization”, “external encapsulating structure”, “collagen type XV trimer”, and “chloride channel activity”, reinforcing the functional interplay between ion channels and ECM dynamics. These findings position ion channels as key regulators of ECM composition, microenvironment remodeling, and metastasis. Additionally, insights from communities 30 and 7 reveal a network of pathways directly or indirectly governed by ion channel activity. Among the most significant are “chenodeoxycholic acid binding”, “extracellular matrix structural constituent”, “ABC-type transporter activity”, “collagen binding”, “ATPase-coupled transmembrane transporter activity”, and “collagen-activated tyrosine kinase receptor signaling”. Collectively, these pathways highlight the roles of Cl^−^ channels and ABC transporters in regulating cell volume, extracellular matrix (ECM) degradation, and metabolite flux, thereby facilitating tumor invasion. Furthermore, ATP-dependent ionic pumps regulate cell adhesion and ECM secretion, underscoring their significant contribution to the interplay between ion transport and tumor microenvironment remodeling (Figure 5).

### 2.7. Statistical External Validation of the Active CRC-IC Module Results

**Figure 6:**
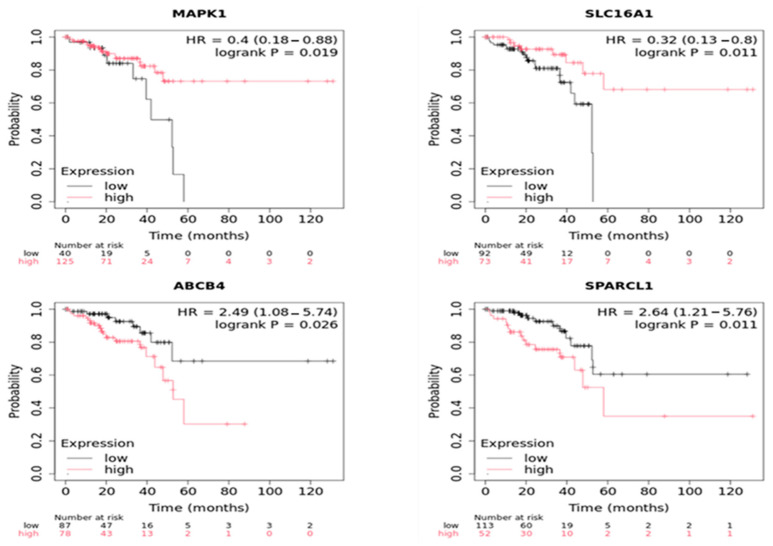
The most relevant genes associated with overall survival (OS) in colon carcinoma. Low and high expression levels are derived (dichotomized) by a ROC analysis.

An external validation procedure was conducted using survival analysis. Kaplan-Meier curves, log-rank tests, and ordinary Cox regression models (on both overall and tumor stage-stratified samples) were employed to assess the impact of differential expression of CRC-IC active module genes on overall survival (OS) in all available CRC samples. The analysis identified several genes whose expression levels were significantly associated with patient prognosis. Among the most notable genes, MAPK1, SLC16A1, SPARCL1, and ABCB4 showed the strongest associations with OS, as demonstrated in Figure 6. Downregulation of MAPK1 is linked to shorter OS, underscoring its role in promoting cell survival and proliferation via the MAPK/ERK signaling pathway. Additionally, downregulation of SLC16A1, a gene encoding the lactate transporter protein, is associated with cytosolic acidification caused by lactate accumulation. In hypoxic tumors, the downregulation of SLC16A1 can be offset by the overexpression of MCT4, which enhances lactate export [28]. The acidic environment, known as the Warburg effect, elevates cytosolic calcium levels and activates key signaling pathways, including the PI3K/AKT/mTOR pathway, which drives tumor growth, metastasis, and therapy resistance, and is linked to poor overall survival (OS), contributing to a significantly worse prognosis. Consequently, the downregulation of SLC16A1 is associated with shorter OS in patients with CRC, highlighting its importance in maintaining normal cellular function and its potential as a prognostic marker. Additionally, SPARCL1, a gene that regulates extracellular matrix (ECM) remodeling and cell adhesion, was significantly correlated with improved survival when expressed at higher levels. In particular, it is involved in type I collagen assembly and decorin deposition, mediating cell adhesion, migration, and proliferation. Its downregulation is associated with several tumors that would otherwise be arrested in the G1 phase of the cell cycle. Thus, it can potentially function as a tumor suppressor in CRC progression. Moreover, ABCB4 expression, which encodes a phospholipid transporter, was associated with OS. The physiological role of the transporter is to safeguard the hepatobiliary system from harmful detergents and lithogenic events [29]. The downregulation of this transporter leads to a proinflammatory environment. It is linked to poorer survival outcomes in CRC, highlighting the critical role of ABCB4 in mediating drug resistance, which could be targeted to improve therapeutic strategies in CRC patients [30].

## 3. Discussion

CRC remains a leading cause of cancer-related mortality, with tumor progression tightly linked to the dynamic interplay between cancer cells and their microenvironment [31]. Among the key molecular determinants of CRC development, ECM remodeling and biomechanical signal transmission play central roles in tumor proliferation, invasion, and metastasis [2]. Emerging evidence highlights the involvement of ion channels in modulating ECM composition, cell adhesion, and mechanotransduction, extending their role beyond the classical functions of ion flux and membrane potential regulation [32,33,34].

As molecular transducers of extracellular mechanical cues, Ion channels are crucial in key cancer hallmarks [10]. Understanding the crosstalk between ion channels and the ECM in CRC may offer novel therapeutic opportunities to disrupt the tumor-supportive microenvironment and enhance treatment efficacy.

This study aimed to unravel the transcriptomic complexity of CRC, focusing on the functional roles of ion channels and their involvement in the desmoplastic tumor microenvironment. A comprehensive gene expression analysis was conducted using a dataset that included 185 CRC and 157 normal adjacent tissue (NAT) samples. This analysis identified 4036 differentially expressed genes (DEGs) (adjusted *p*-value < 0.01), of which 1986 were upregulated (log2 fold change ≥ 1) and 2050 were downregulated (log2 fold change ≤ −1).

Intersecting these DEGs with a curated list of ion channel–encoding genes revealed 183 ion channels that exhibited significant differential expression. Among them, 113 genes were downregulated, including *ABCC8*, *CLCA1*, *KCNMA1*, *TRPM4*, *TRPM5*, *TRPM6*, *TRPV3*, *SCN2B*, *SCN3A*, *SCN3B*, *SCN4B*, *SCN7A*, *SCN9A*, *SCNN1B*, and *SCNN1G*. Conversely, 70 genes were upregulated, including *ABCC9*, *CLCN4*, *CLDN16*, *CLDN2*, *KCNH8*, *KCNJ11*, *KCNJ14*, *KCNJ15*, *KCNN4*, and *TRPV4*.

The identified genes encode diverse ion channel subunits, including voltage-gated, calcium-gated, ligand-gated, and mechanosensitive channels, underscoring the potential role of ion channel dysregulation in CRC pathophysiology. These channels mediate various ion transport and signaling mechanisms highly relevant to cancer biology. Our results align with previous reports showing the dysregulation of TRP channels in CRC, such as elevated TRPV4 and reduced TRPA1 levels [35], further supporting their prognostic relevance. Additionally, potassium and calcium channels appear critical in maintaining intracellular Ca^2+^ homeostasis, which is modulated by lipids, proteins (e.g., STIM), receptors (e.g., S1R/SIGMAR1), and peptides (e.g., LL-37)—offering novel therapeutic targets through monoclonal antibody modulation [36].

The dataset showed strong concordance with the TCGA data, with an 85% overlap in DEGs. This substantial agreement reinforces the reliability of our results while recognizing that the remaining 15% variation likely reflects inherent biological diversity, including population-specific genetic backgrounds, epigenetic landscapes, tumor microenvironment heterogeneity, and methodological differences between sequencing platforms and analytical pipelines.

Following this validation step, we conducted a Reactome pathway analysis to investigate the relationship between altered ion channel activity and ECM remodeling in CRC. The analysis revealed 396 significantly enriched pathways (*p* < 0.05), with robust representation of ECM-related processes. These include collagen fibril assembly and multimeric structure formation, collagen degradation and biosynthesis pathways, non-integrin-mediated membrane-ECM interactions, and ECM proteoglycan networks. Similar findings have been reported by Li. Y et al. [37], who also observed disrupted collagen pathways associated with microbial shifts, including increased Sutterella, Collimonas, and Campylobacter species.

We integrated Reactome pathways from our ion channel gene set with GEO data through network analysis to construct a comprehensive interaction map comprising 581 molecular nodes and 602 edges. Further network analysis revealed a functionally coherent subnetwork (Figure 4) demonstrating the dynamic interplay between ECM components, ion channel function, and cytoskeletal organization in CRC pathogenesis.

Our study observed a marked downregulation of TRPM5, a monovalent intracellular calcium-dependent cation channel [38]. CRC shows a different profile for this channel, unlike other tumors, in which TRPM5 activation is related to acidic pH and MMP9-mediated extracellular matrix remodeling. Our data indicate that TRPM5 suppression promotes CRC progression. In particular, reduced TRPM5 activity leads to an increase in cytosolic Ca^2+^ concentration, which stimulates proliferative pathways and the production of inflammatory cytokines such as IL-6 and CXCL10, with particularly marked effects at the invasive edges of the tumor [39]. The increase in IL-6 is clinically relevant, as it is associated with advanced stages of tumors and a poor prognosis. Moreover, it may contribute to T-cell resistance to apoptosis, a phenotype observed in chronic inflammatory bowel diseases (IBD), through BCL-2 activation [40,41]. TRPM5 downregulation is also associated with reduced PLCG2 expression, which contributes to intracellular Ca^2+^ accumulation and activates proliferative pathways by increasing p-AKT and Cyclin D1 levels [41]. Dynamic interactions with the tumor microenvironment shape this molecular scenario. For instance, a high expression of CHST11 (carbohydrate sulfotransferase 11), an enzyme that sulfate glycosaminoglycans such as chondroitin sulfate, a key component of the extracellular matrix, has been observed. CHST11 activity promotes the accumulation of versican (VCAN), a multifunctional proteoglycan involved in both stromal remodeling and immune evasion. Proteolytic degradation of VCAN by MMPs and ADAMTS produces bioactive fragments, known as matrikines, including versikine, which contributes to the creation of an immunomodulatory environment influencing the migration of CD8^+^ lymphocytes and promoting immune evasion of the tumor [42,43,44,45,46]. Beyond ion channel alterations, our study revealed significant dysregulation of key fibrous ECM components in CRC, with notable overexpression of collagen genes (*COL5A2*, *COL1A1*, *COL4A1*, *COL6A3*, and *COL15A1*) and laminin subunits (*LAMA5* and *LAMC1*) [47]. These changes occurred in concert with increased PCOLCE (procollagenase C-proteinases) enzymes that remove C-terminal peptides from procollagen, forming and depositing mature collagen fibers. Expression and altered ADAMT activity are critical regulators of collagen processing and ECM deposition in the heart. The functional implications of these changes are complex. PCOLCE upregulation, potentially linked to TRPM5 suppression, drives collagen accumulation and proliferative signaling. At the same time, ADAMTs and metalloproteinases actively process immature procollagens (types I, III, and V) to promote extracellular matrix remodeling.

Particularly noteworthy is the strong stromal expression of *COL4A1*, *COL6A3*, and *COL1A1* in CRC, which multiple studies have associated with adverse clinical outcomes, suggesting their potential utility as stromal-derived prognostic markers [47]. The tumor-promoting effects of COL1A1 may be partly mediated through its activation of the WNT/PCP signaling pathway, which stimulates JNK-dependent cytoskeletal reorganization—a known driver of invasive progression [48].

Characterization of the CRC–(IC) module offers critical insights into the molecular interactions that govern tumor microenvironment dynamics. Enriching gene clusters involved in ECM remodeling, ion transport, and mechanotransduction underscores their functional significance in CRC progression.

Furthermore, key prognostic markers, such as *MAPK1* and *SLC16A1*, were identified, with altered expression correlating with patient survival [49,50]. The association between *ABCB4* expression and drug resistance suggests that targeted modulation of ion transport mechanisms may help overcome therapy resistance in CRC.

While our study provides valuable insights, it is limited by its reliance on transcriptomic data, which do not capture post-translational modifications or protein activity. Additionally, validation across diverse populations is required to confirm the generalizability of these findings. Future functional studies, including gene knockdown and pharmacological inhibition [31], are crucial for validating the therapeutic potential of targeting ion channel–ECM interactions in CRC. For these reasons, functional validation of the study is currently underway through in vitro experiments.

Due to the novelty of this research field, experimental studies (both in vitro and in vivo) are limited. However, some studies have highlighted how activating specific channels, such as TRPV4 or TRPM5, leads to extracellular matrix remodeling in tumors and desmoplastic conditions [16] through calcium signaling. For example, the high intracellular calcium concentration resulting from TRPM5 activity enhances matrix metalloproteinase-9 (MMP-9) in tumors, which promotes ECM remodeling and contributes to cancer progression [39]. Furthermore, TRPV4 is implicated in collagen synthesis and remodeling [16]. Our research is investigating ion channel−ECM interactions to understand the role of mechanosensing in tumor progression and to identify potential new therapeutic targets in the TME.

The study has limitations: The analysis pipeline cannot fully capture protein-level activity or post-translational modifications. Additionally, because we used a single ethnic cohort (Korean), the generalizability of the results to broader populations regarding the understanding of CRC mechanisms across different genetic backgrounds may be limited. Furthermore, a key limitation of our study is the relatively small sample size when compared to the statistical external validation, which restricted our ability to stratify patients by clinical variables, such as tumor stage, without compromising statistical power. This limitation underscores the necessity for future studies with larger sample sizes to validate and extend our findings.

## 4. Materials and Methods

### 4.1. Data Collection and Study Design

**Figure 7:**
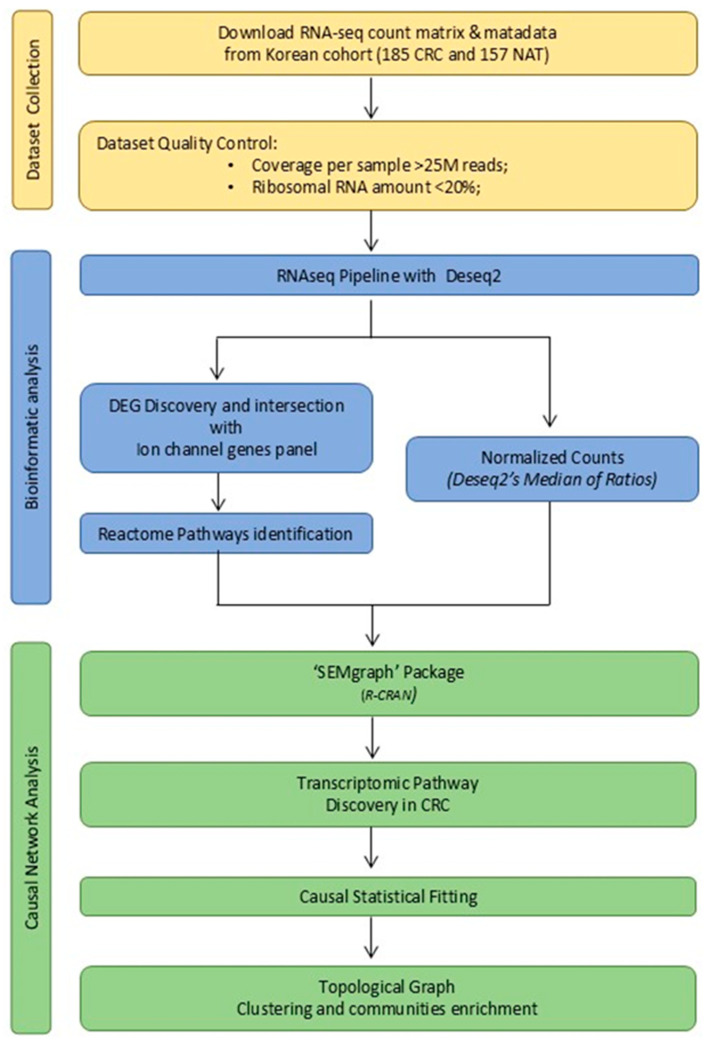
The study workflow. The dataset selection step is highlighted in yellow, RNA-seq bioinformatics analysis in light blue, and causal network analysis step is highlighted in green.

To identify the CRC-IC active module and cluster communities, a large-scale RNA-seq raw count matrix and the corresponding patient metadata from a Korean cohort, as described by Lee J. et al. [27], were retrieved from Zenodo https://zenodo.org/records/8333650 (accessed on 12 February 2025). The complete workflow is illustrated in Figure 7.

### 4.2. Dataset Quality Control and Ion-Channel Gene Panel Selection

Since achieving 20–30 million reads per sample is essential for comprehensive coverage, accurate quantification of gene expression, and detection of low-abundance transcripts in RNA-seq analysis of complex eukaryotic transcriptomes [51], a quality control threshold of 25 million reads was established. The complete pipeline utilized for full quality control is available at https://github.com/MichelangeloAloisio/Transciptomic_disease_module_in_CRC (accessed on 30 March 2025).

To select the ion-channel gene panel, the NCBI Gene database (https://www.ncbi.nlm.nih.gov/gene/ (accessed on 3 March 2025) was queried using the following search term: (“Homo sapiens” [Organism] and “channel” [GO]). This query retrieves genes annotated with the term ‘channel’ in the Gene Ontology for Homo sapiens.

### 4.3. IC-DEGs Identification and Normalized Counts Production

To assess data variability before differential expression analysis, Principal Component Analysis (PCA) was performed on log-transformed count data, focusing on the top 500 most variable features, using the default settings of DESeq2 [52]. Sex, disease status and grade were evaluated as potential contributors to the observed variations. Normalized counts were generated using the median-of-ratios method, retaining genes with >10 counts in at least 10 samples. These normalized counts were crucial for downstream analyses, including causal network construction, which relies on robust normalization to model the gene interactions accurately. Differential expression analysis was performed by comparing CRC patients to healthy controls using a negative binomial generalized linear model in DESeq2 [52]. DEGs were defined as genes with |fold change| ≥ 2 and an adjusted *p*-value < 0.01, and were visualized using volcano plots. The script for this analysis has been described previously [23]. To identify differentially expressed ion-channel genes (IC-DEGs), a Venn diagram was created using Venny 2.1 available at the link https://bioinfogp.cnb.csic.es/tools/venny/index.html (accessed on 5 March 2025) [53], which intersected DEGs with the preselected ion-channel gene panel. This intersection identified significantly dysregulated ion channel genes in patients with CRC relative to healthy controls.

### 4.4. IC-DEGs External Validation

To validate the expression patterns of IC-DEGs in normal and tumor tissues identified in our dataset, we performed a comprehensive analysis incorporating 56,938 unique samples from the Gene Expression Omnibus (GEO) available at the link https://www.ncbi.nlm.nih.gov/geo/ (accessed on 7 March 2025) [54], Genotype-Tissue Expression (GTEx) available at the link [55], The Cancer Genome Atlas (TCGA) [56]. Therapeutically Applicable Research to Generate Effective Treatments (TARGET) [57] databases. These samples included 15,648 from normal tissues, 40,442 from tumors, and 848 from metastatic tissue.

We utilized the KM Plotter [58], a robust tool for survival biomarker discovery and validation, to perform differential expression analysis, patient survival analysis, correlation profiling, and other customizable analyses. The gene expression plots generated using KM Plotter were based on clinical annotations from TCGA, ensuring accuracy and relevance.

To further validate our findings at the translational level, we used the Human Protein Atlas database, which provided protein-level confirmation of the channel-coding genes identified in our dataset. This integrative approach ensured robust IC-DEG validation, underscoring their biological significance and potential translational impact on cancer research.

### 4.5. Reactome Enrichment Analysis

The selected IC-DEGs, identified by their gene symbols, were subjected to pathway enrichment analysis using the Reactome Pathway Analysis Tool (v88) [Reactome Analysis Tool, available online: https://reactome.org/PathwayBrowser/#TOOL=AT, accessed on 5 November 2024]. Pathways with a false discovery rate (FDR) < 95% were considered relevant and selected for further analysis. Specifically, the FDR was used as a metric to involve the highest possible number of pathways (by Reactome Pathways, https://reactome.org/, accessed on 5 November 2024, [59]), by accounting for the trade-off between complexity, perturbation, and statistical fitting [28].

In the Reactome tool, the following options were enabled: projection to human genes and including interactors. This analysis generated a list of pathways involving at least one IC-DEG, which was subsequently used in the R/SEMgraph packages to build the network [28].

### 4.6. Statistical Analysis

To identify and model the ion channels (IC) in the colorectal cancer active module (network), we employed a combination of graph theory and structural equation modeling (SEM). Graph theory is widely used to model complex biological networks and has numerous applications in medical and biological research involving omics data [24,25]. SEM is a multivariate statistical approach that employs simultaneous equations to describe the relationships between variables. In this framework, each variable (node or gene) can serve as both an explanatory variable in some equations and an outcome variable in others [26].

Our network was constructed using notable genes, excluding newly discovered transcripts from RNA-seq analysis. The process involved two main steps: (1) SEM-based gene set analysis (SEMgsa) [28] was performed to identify differential expression patterns, and (2) the active CRC-IC module was fitted based on these patterns. Graph theory was applied to (i) learn the causal architecture using Reactome data [59] to define the active CRC-IC module and (ii) identify network communities and paths to explore structural relationships within the network. SEM was used to (i) perform initial differential expression analysis (SEMgsa) (Supplementary Table S14) and (ii) refine the active CRC-IC module through model fitting. Notably, graph theory also plays a critical role during the model searching phase of the fitting process.

#### 4.6.1. Analysis of the Active CRC-IC Module

We constructed and fitted an SEM-based transcriptomic active CRC-IC module using differentially expressed genes (DEGs) identified through SEMgsa analysis (details are provided in the Supplementary Files). These DEGs, called seed genes, were initially mapped onto the Reactome interactome, representing the union of all Reactome pathways, using a graph-weighting procedure based on Fisher’s r-to-z transformation [60]. This approach tested the differences in the correlation coefficients of interacting gene pairs between groups [61]. To generate a perturbed reduced graph representing the active CRC-IC module, we applied a Steiner tree (ST) identification procedure based on Kou’s algorithm [62]. This process connected seed genes to other genes (connector genes) by minimizing the total edge distance. The ST procedure transformed *p*-values from the r-to-z Fisher transformation into the inverse of the negative log(*p*-value), ensuring positive, continuous edge weights. In graph theory, the ST is an acyclic graph that connects seed genes to additional nodes in the most compact manner [28].

After constructing the transcriptomic active CRC-IC module, SEM was used to quantify and verify perturbations (status group → gene) and effects (gene-i → gene-j), denoted as β, among genes within the module by comparing different groups [28]. Statistical significance was assessed using two-tailed z-tests with the null hypothesis H0: β = 0. Significance was defined as an adjusted *p*-value of <0.05 [23,26,59,63]; specifically, *p*-values were derived by applying Benjamini-Hochberg multiple test corrections within DAG-based procedures (i.e., de-confounding and model adjustment) [28].

SEM fitting utilized constrained Gaussian graphical modeling (CGGM), incorporating a DAG node-wise Lasso procedure and debiased asymptotic inference [64].

The effects of gene-i or group perturbations on gene-j are represented as conditionally interpretable regression coefficients. These coefficients reflect the expected normalized change in gene-j expression per unit increase in gene-i expression (or the difference between cases and controls), while holding other variables constant within the module. Negative coefficients indicate inhibition, while positive coefficients indicate activation. Similarly, negative group effects indicated downregulation, and positive values indicated upregulation [63]. Three models were fitted: (i) Model A evaluated the edge effects specifically in CRC data. (ii) Model B evaluated the edge effects in controls (NAT). (iii) Model C evaluated both node and edge perturbation effects.

For Model C, a two-step procedure was conducted: (1) The group was modeled as an exogenous variable influencing all other graph nodes. (2) Differences in beta coefficients (edges) between groups were estimated, similar to fitting separate models for cases and controls and assessing the edge significance based on these differences.

Indirect effects were examined to identify potential gene mediators. To improve SEM fitting, strategies included extracting the optimal Directed Acyclic Graph (DAG) by balancing model adjustment and graph sparsity, and implementing a de-confounding process to minimize badness-of-fit measures while maintaining strong perturbation signals [28]. Beta coefficients were relaxed to establish a trade-off between model fit and biological relevance, with tuning achieved by incrementing values from 0.05 to 0.15 in 0.01 steps.

We aimed to optimize the SEM fitting by balancing the model complexity, fit, and perturbation. A goodness-of-fit index, such as the standardized root mean square residual (SRMR), was calculated to assess the model fit [60]. While this study is exploratory, an SRMR < 0.10 is generally considered adequate, and values < 0.05 indicate a good fit [65].

#### 4.6.2. Gene Communities Detection

A clustering procedure was performed to identify topological gene communities using the walk-trap community detection algorithm (WTC) [28,66]. This algorithm generates clusters encompassing the entire input network. A modularity index was calculated to evaluate the effectiveness of clustering, which measures the degree of separation between communities; values close to 1 indicate well-defined community structures. Clustering was applied to both the Steiner tree graph and the optimal Directed Acyclic Graph (DAG) obtained from the previously described fitting strategies, allowing for comparing the outcomes. Statistical analyses were conducted using R software (version 4.3.3, R Core Team, 2023) with the SEMgraph [28], huge [60], and hgu95av2.db packages (R code provided in the Supplementary Materials). The R/SEMgraph package models complex biological systems as causal multivariate networks, while the R/huge package performs non-paranormal transformations of gene expression data. The R/hgu95av2. The db package was used for annotation, converting Entrez identifiers into gene symbols for the graphical representation of the networks.

### 4.7. Communities’ Enrichment Analysis

Functional enrichment analysis was performed using the gProfiler bioinformatics tool [67] to investigate the biological characteristics of the 36 communities identified through the topological clustering of the active CRC-IC module. The analysis queried the Gene Ontology (GO) databases, focusing on Molecular Function, Biological Processes, and Cellular Components, to provide comprehensive insights into these communities’ functional roles and biological relevance. Multiple testing corrections were applied using the g:SCS algorithm [67].

### 4.8. Statistical External Validation of the Active CRC-IC Module

For the most representative genes within the Active CRC-IC module, survival analyses (i.e., Kaplan-Meier curves and Cox regression modeling) were performed using the online platform KM Plotter (A5 Genetics Ltd., Und, Hungary) [58]. This analysis was based on clinical and transcriptomic data from The Cancer Genome Atlas (TCGA) [56] for colorectal adenocarcinoma. By associating gene expression levels with survival outcomes, this approach identified key genes potentially associated with disease prognosis, both overall and stratified by tumor stage.

## 5. Conclusions

This study elucidates the transcriptomic interplay between ion channels and the ECM in CRC, revealing a network of dysregulated pathways that drive tumor progression. By integrating RNA-seq data with causal network inference, we identified 188 ion channel-associated differentially expressed genes (DEGs) enriched in ECM remodeling processes, including collagen deposition, matrix degradation, and mechanosignaling. The CRC-IC module identified critical nodes, such as *TRPM5*, whose downregulation disrupts calcium signaling and promotes proliferation, and *CHST11*, which enhances versican-mediated immune evasion. Overexpression of collagen genes (*COL1A1* and *COL5A2*) and dysregulation of ECM enzymes further highlight the stromal contribution to tumor aggression.

**Figure 8:**
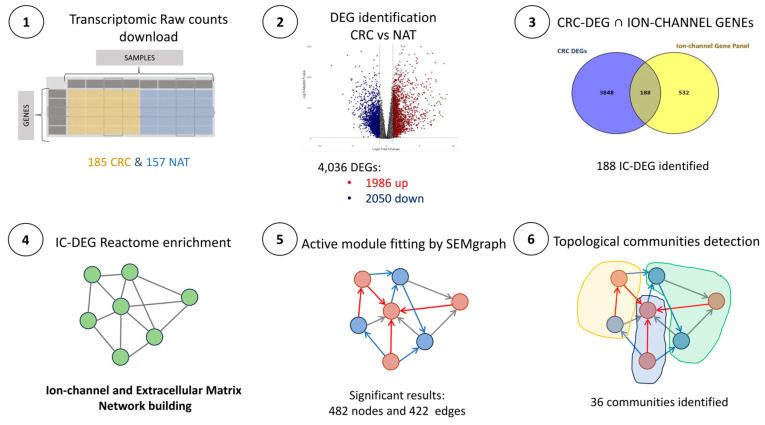
Conceptual workflow. The box (4) shows the network (i.e., the active CRC-IC module) achieved by the graph-based algorithms, from the DEG Reactome enrichment, before the SEM fitting. The boxes (5) and (6) show an active CRC-IC module, red dots represent overexpressed genes, while blue dots indicate down-expressed genes. Red arrows denote significant direct effects with a positive association, and blue arrows indicate significant direct effects with a negative association.

Survival analysis of *MAPK1* and *SLC16A1* highlights their prognostic relevance, while the downregulation of *ABCB4* is associated with poor outcomes, suggesting its involvement in drug resistance mechanisms. Our network-driven approach bridges molecular heterogeneity with micro-environmental dynamics, positioning ion channels as central players in CRC pathophysiology (see Figure 8). Future studies should experimentally validate these targets and explore therapeutic strategies to disrupt ion channel−ECM feedback loops, potentially mitigating metastasis and therapy resistance in CRC. 

## Figures and Tables

**Table 1:** Age, sex, and disease stage in the Korean Cohort.

Category	Value
CRC Sample Number	185
NAT Sample Number	157
Age (mean ± SD)	62.3 ± 10.5 years
Sex	Male: 112 (60.5%), Female: 73 (39.5%)
Disease Grade	Grade I: 22 (11.9%), Grade II: 58 (31.4%), Grade III: 75 (40.5%), Grade IV: 30 (16.2%)

**Table 2:** Relations between the community and graph-reduced genes.

Community_ID	Community Gene Number	Intersection with Reduced Graph	Intersection Percentage
35	5	5	100
36	5	4	80
19	13	8	62
29	5	3	60
30	6	3	50
7	66	32	48
20	10	2	20
4	48	1	2

## Data Availability

The data presented in the study are openly available at https://dx.doi.org/10.6084/m9.figshare.28715540 (accessed 20 March 2025).

## Supplementary Materials

The following supporting information can be downloaded at: https://dx.doi.org/10.6084/m9.figshare.28715540 (accessed 20 March 2025).
